# Dynamic nutritional trajectories and deterioration risk in esophageal cancer radiotherapy: a prospective study

**DOI:** 10.3389/fnut.2026.1828784

**Published:** 2026-06-18

**Authors:** Jiayi Chen, Cheng Feng, Xinmei Zhang, Di Qin, Lihong Zhang, Qi An, Siyuan Qu, Tianyu Li, Jun Liang, Meng He

**Affiliations:** 1 Department of Radiation Oncology, Shenzhen Hospital, National Cancer Center/Cancer Hospital, Chinese Academy of Medical Sciences and Peking Union Medical College, Shenzhen, Guangdong, China; 2 School of Medicine, The Chinese University of Hong Kong, Shenzhen, China; 3 Department of Thyroid and Galactophore Surgery, People’s Hospital of Longhua, Shenzhen, China

**Keywords:** esophageal cancer, nutritional deterioration, PG-SGA, radiotherapy, sleep disturbance

## Abstract

**Background:**

Nutritional deterioration (ND) during radiotherapy (RT) for esophageal cancer is common, yet the optimal timing for proactive supportive care remains unclear. We characterized time-resolved nutritional trajectories, examined baseline factors associated with deterioration, and translated the findings into a patient-facing digital prototype.

**Methods:**

In this prospective longitudinal cohort, 123 patients underwent nutritional assessment at five timepoints (admission, RT1, RT14, RT20, and discharge) using the Patient-Generated Subjective Global Assessment (PG-SGA), serum albumin, and total protein. ND was defined as an upward shift in PG-SGA risk category (0–3/4–8/≥9) from admission to discharge. Sleep was assessed by baseline interview, the Pittsburgh Sleep Quality Index (PSQI), and exploratory wearable measures. Logistic regression was used to identify baseline factors associated with ND. Complementary continuous-change analysis and linear mixed-effects modeling were additionally performed to characterize deterioration magnitude and longitudinal trajectories. Exploratory correlations assessed sleep–nutrition associations. Based on the findings, a digital prototype (“Esophageal Care Companion”) was developed with risk-aware onboarding and phase-specific monitoring prompts.

**Results:**

PG-SGA increased from 4.59 ± 3.66 at admission to 6.95 ± 3.48 at RT20 (*p* < 0.001), with concordant declines in albumin and total protein. ND occurred in 40.7% (50/123). In the primary categorical model, baseline sleep problems were associated with higher odds of ND [adjusted odds ratio (aOR) 2.48; *p* = 0.034], as was N3 nodal stage (aOR 3.56; *p* = 0.022). In complementary analyses, N3 remained associated with greater PG-SGA worsening and a distinct longitudinal nutritional trajectory, whereas the sleep signal was less consistent across continuous and longitudinal models. Sleep measures showed exploratory phase-specific associations with nutritional biomarkers, including RT14 rapid eye movement sleep duration with albumin (*r* = 0.258; *p* = 0.004) and discharge PSQI with PG-SGA (*r* = 0.276; *p* = 0.002).

**Conclusion:**

Nutritional risk worsened during esophageal cancer RT, particularly in the mid-to-late treatment phase, supporting RT14–RT20 as a clinically relevant window for intensified assessment and supportive-care escalation. Advanced nodal stage emerged as a robust marker of less favorable nutritional trajectory. Baseline sleep problems may serve as a low-burden observational risk signal but should be interpreted cautiously. The prototype demonstrates the feasibility of translating phase-aware risk monitoring into digital supportive care.

## 1 Introduction

Esophageal cancer remains a major global malignancy with substantial mortality, and China bears a high disease burden (1, 2). Radiotherapy, often combined with chemotherapy, is a cornerstone treatment for many patients, particularly those with locally advanced disease or who are not surgical candidates (3). Despite advances in supportive care, nutritional compromise during radiotherapy remains common and clinically consequential (4, 5).

Radiotherapy-related toxicities—particularly acute radiation esophagitis—typically develop during the course of treatment and may worsen as the delivered dose accumulates. This results in odynophagia/dysphagia, reduced oral intake, and symptom-driven dietary restriction (6, 7). Malnutrition and deteriorating nutritional status are associated with poorer treatment tolerance, higher complication risk, and potential treatment disruptions, highlighting the importance of timely assessment and phase-appropriate nutritional intervention (8, 9).

However, evidence informing when and for whom nutritional surveillance should be intensified during radiotherapy remains limited (10). Most available reports evaluate nutritional status at only one or two time points (baseline and post-treatment), limiting the characterization of within-patient trajectories or the identification of clinically interpretable “vulnerability windows” (11, 12). Furthermore, sleep disturbance is highly prevalent during cancer treatment (13), frequently accompanied by greater symptom burden and impaired functioning (14), raising the possibility that sleep disruption signals heightened nutritional vulnerability (15). Yet, the relationship between sleep disturbance and clinically meaningful nutritional deterioration specifically during esophageal cancer radiotherapy remains insufficiently characterized.

Therefore, we conducted a prospective longitudinal cohort study with repeated assessments to characterize within-patient nutritional trajectories and examine baseline factors associated with nutritional deterioration during treatment. Nutritional status was assessed using the Patient-Generated Subjective Global Assessment (PG-SGA) and supplemented by serum biomarkers (16). Nutritional deterioration (ND) was defined as an upward shift in PG-SGA risk category from admission to discharge. We evaluated baseline clinical factors, including sleep problems, in relation to ND and explored associations between sleep measures and nutritional indicators (17, 18).

## 2 Materials and methods

### 2.1 Study design and participants

This prospective longitudinal cohort study was conducted in the Department of Radiation Oncology, Shenzhen Hospital of the National Cancer Center, from October 2021 to November 2023 (Ethics Approval: KYKX201815). All participants provided written informed consent.

Consecutive adult patients with pathologically confirmed esophageal cancer receiving radiotherapy were screened. Inclusion criteria were: (1) age ≥18 years; (2) pathologically confirmed esophageal cancer; (3) receiving radiotherapy; (4) ability to complete assessments; and (5) estimated survival >6 months. Exclusion criteria were systemic autoimmune disorders, severe vital organ dysfunction, inability to complete questionnaires, substance abuse history, severe respiratory disease, or primary nutritional disorders. A total of 123 patients were included.

### 2.2 Measures and data collection

Baseline clinical and demographic variables were collected at admission, including anthropometrics, Nutritional Risk Screening 2002 (NRS 2002) (19), Karnofsky Performance Status (KPS) (20), comorbidities, baseline pain, sleep status, tumor characteristics, and concurrent chemoradiotherapy status. Nutritional and sleep-related measures were assessed at prespecified radiotherapy timepoints.

#### 2.2.1 Nutritional status assessment

##### 2.2.1.1 NRS 2002

Baseline nutritional risk was screened at admission (19).

##### 2.2.1.2 PG-SGA

Nutritional status was assessed using the scored Patient-Generated Subjective Global Assessment (PG-SGA) at five prespecified timepoints: admission, first fraction (RT1), 14th fraction (RT14), 20th fraction (RT20), and discharge (21). PG-SGA scores were operationalized into three numerical risk strata: 0–3 (low risk), 4–8 (moderate risk), and ≥9 (high risk) (16, 21).

##### 2.2.1.3 Primary endpoint: nutritional deterioration

ND was defined *a priori* as an upward shift in PG-SGA numerical risk stratum from admission to discharge (0–3 → 4–8 or ≥9; 4–8 → ≥9). All other patterns were classified as non-deterioration (stable/improved). This categorical endpoint was prespecified for clinical interpretability in supportive-care triage. Because it may not fully capture change magnitude and may be constrained by baseline ceiling effects, complementary continuous-change and longitudinal analyses were additionally performed.

##### 2.2.1.4 Laboratory biomarkers and anthropometrics

Serum albumin, total protein, body weight, and BMI were collected per clinical workflow and aligned to prespecified timepoints.

### 2.2.2 Sleep assessment

#### 2.2.2.1 Baseline sleep status

Assessed via nurse interview at admission referring to the preceding week. Status was categorized primarily as a binary variable (any sleep problem vs. normal) for robustness in regression modeling.

#### 2.2.2.2 Wearable-derived sleep metrics

Objective metrics were collected using a consumer wearable device (Xiaomi Band), generating summaries aligned to prespecified timepoints. These were analyzed as exploratory measures (18).

#### 2.2.2.3 PSQI

Subjective sleep quality was assessed using the Pittsburgh Sleep Quality Index (PSQI) at RT1, RT14, RT20, and discharge (17).

### 2.2.3 Radiotherapy regimen and supportive care

Information on radiotherapy regimen and treatment-related supportive care was abstracted from medical records. Extracted radiotherapy variables included total dose, dose per fraction, and total number of fractions. Nutritional support variables included any nutritional support, oral nutritional supplementation, nutrition consultation, diet modification or texture adjustment, nasogastric tube feeding, post-pyloric tube feeding, gastrostomy, and parenteral nutrition. Analgesic support to facilitate oral intake was also recorded as a related supportive-care measure. These measures were delivered according to routine clinical practice rather than a prespecified standardized nutritional protocol.

### 2.3 Statistical analysis

Analyses were performed in SPSS v26.0, and two-sided *p* < 0.05 was considered statistically significant. Descriptive data are presented as mean ± standard deviation (SD) or n (%), as appropriate.

Longitudinal changes in PG-SGA score, serum albumin, and total protein across the five prespecified timepoints were first evaluated using repeated-measures ANOVA with Greenhouse–Geisser correction. Univariable and multivariable logistic regressions were then used to identify baseline predictors of the primary categorical endpoint, ND. Prespecified covariates in the multivariable models included age, NRS 2002 score, chronic disease, baseline sleep problems, and advanced nodal stage (N3 vs. N0–2).

To complement the categorical endpoint, continuous change in PG-SGA from admission to discharge was additionally analyzed as ∆PG, defined as discharge PG-SGA minus admission PG-SGA. Group differences in ∆PG were compared using independent-samples t tests, and multivariable linear regression was used to examine baseline factors associated with greater PG-SGA worsening. The same prespecified covariates (age, NRS 2002 score, chronic disease, baseline sleep problems, and advanced nodal stage) were included in the linear regression model.

Sensitivity analysis was performed by excluding patients with baseline PG-SGA ≥ 9 to reduce potential ceiling effects in the categorical ND definition. The multivariable logistic regression model was then repeated in this restricted sample.

To further characterize repeated-measures trajectories, linear mixed-effects modeling was performed with repeated PG-SGA score as the dependent variable. Fixed effects included timepoint, baseline sleep problems, advanced nodal stage, age, NRS 2002 score, and chronic disease. A first-order autoregressive [AR(1)] covariance structure was specified for repeated observations to account for within-patient correlation. Additional interaction models included time-by-sleep and time-by-nodal-stage terms to evaluate whether longitudinal nutritional trajectories differed by these baseline factors.

Exploratory associations between sleep measures and nutritional indicators were assessed using Pearson correlations.

### 2.4 Digital prototype development

To translate our findings into a patient-support pathway, we developed a digital prototype (“Esophageal Care Companion”) using Google AI Studio. Development was informed by evidence supporting electronic patient-reported outcomes and remote symptom monitoring during cancer treatment (22, 23). The prototype incorporated structured symptom-question design principles (e.g., PRO-CTCAE) and pragmatic engagement considerations, and presented exploratory sleep–nutrition associations as non-causal insights (24–26). Only synthetic, non-identifiable inputs were used during testing.

## 3 Results

### 3.1 Baseline characteristics

Of the 123 patients, 73 (59.3%) remained stable/improved and 50 (40.7%) experienced ND. Baseline demographic characteristics, including age, sex, BMI, and NRS 2002, were comparable between groups (Table 1). However, patients with ND had significantly higher rates of baseline sleep problems (42.0% vs. 24.7%; *p* = 0.042) and N3 nodal disease (26.0% vs. 8.2%; *p* = 0.007). Comorbidity status and admission PG-SGA scores also differed significantly between the groups. Admission PG-SGA was lower in the ND group under this categorical worsening definition, suggesting possible baseline severity constraint in the stratified endpoint. Radiotherapy regimens were heterogeneous but were predominantly delivered using conventional fractionation, most commonly 50 Gy in 25 fractions (67/123, 54.5%) and 60 Gy in 30 fractions (23/123, 18.7%). Nutritional support during treatment was common: 99/123 patients (80.5%) received at least one form of nutritional support, most frequently oral nutritional supplementation (90/123, 73.2%) and diet modification or texture adjustment (94/123, 76.4%). In addition, 23 patients (18.7%) received nasogastric tube feeding, 5 (4.1%) received post-pyloric tube feeding, 1 (0.8%) underwent gastrostomy, and 34 (27.6%) received parenteral nutrition. Analgesic support to facilitate oral intake was also commonly documented as a related supportive measure (53/123, 43.1%). Detailed data are provided in Supplementary Table S1.

**Table 1:** Baseline demographic characteristics.

Characteristic	Total (*N* = 123)	Stable/improved (*n* = 73)	Nutritional deterioration (*n* = 50)	*p*-value
Demographics				
Age, years	63.25 ± 9.53	62.45 ± 9.66	64.42 ± 9.30	0.262
BMI, kg/m²	21.26 ± 3.26	20.85 ± 3.49	21.87 ± 2.81	0.090
NRS 2002 score	1.84 ± 1.21	2.01 ± 1.32	1.60 ± 1.01	0.051
KPS score	86.95 ± 15.10	87.33 ± 12.08	86.40 ± 18.79	0.739
PG-SGA at admission	4.59 ± 3.66	5.90 ± 4.03	2.68 ± 1.78	<0.001
Clinical characteristics				
Sex, n (%)				0.665
Male	96 (78.0)	56 (76.7)	40 (80.0)	
Female	27 (22.0)	17 (23.3)	10 (20.0)	
Chronic disease, n (%)				0.043
No	53 (43.1)	26 (35.6)	27 (54.0)	
Yes	70 (56.9)	47 (64.4)	23 (46.0)	
Pain, n (%)				0.085
No	88 (71.5)	48 (65.8)	40 (80.0)	
Yes	35 (28.5)	25 (34.2)	10 (20.0)	
Concurrent chemoradiotherapy, n (%)				0.813
No	55 (44.7)	32 (43.8)	23 (46.0)	
Yes	68 (55.3)	41 (56.2)	27 (54.0)	
Sleep status (5-level), n (%)				0.037
Normal	84 (68.3)	55 (75.3)	29 (58.0)	
Difficulty falling asleep	16 (13.0)	8 (11.0)	8 (16.0)	
Easy awakening	14 (11.4)	6 (8.2)	8 (16.0)	
Early awakening	5 (4.1)	4 (5.5)	1 (2.0)	
Dreaminess	4 (3.3)	0 (0.0)	4 (8.0)	
Sleep status (binary), n (%)				0.042
Normal	84 (68.3)	55 (75.3)	29 (58.0)	
Any sleep problem	39 (31.7)	18 (24.7)	21 (42.0)	
Sleep-aid use (3-level), n (%)				0.232
None	46 (37.4)	28 (38.4)	18 (36.0)	
Long-term	70 (56.9)	43 (58.9)	27 (54.0)	
Occasional	7 (5.7)	2 (2.7)	5 (10.0)	
Tumor characteristics				
Tumor location (5-level), n (%)				0.838
Upper thoracic	16 (13.0)	8 (11.0)	8 (16.0)	
Middle thoracic	46 (37.4)	30 (41.1)	16 (32.0)	
Lower thoracic	39 (31.7)	22 (30.1)	17 (34.0)	
Gastroesophageal junction	14 (11.4)	8 (11.0)	6 (12.0)	
Other	8 (6.5)	5 (6.8)	3 (6.0)	
Clinical T category (0–4), n (%)				0.052
T1	16 (13.0)	7 (9.6)	9 (18.0)	
T2	13 (10.6)	4 (5.5)	9 (18.0)	
T3	74 (60.2)	49 (67.1)	25 (50.0)	
T4	20 (16.3)	13 (17.8)	7 (14.0)	
Clinical N category (0–3), n (%)				0.007
N0	23 (18.7)	11 (15.1)	12 (24.0)	
N1	21 (17.1)	12 (16.4)	9 (18.0)	
N2	60 (48.8)	44 (60.3)	16 (32.0)	
N3	19 (15.4)	6 (8.2)	13 (26.0)	
Clinical N category (binary), n (%)				0.007
N0-2	104 (84.6)	67 (91.8)	37 (74.0)	
N3	19 (15.4)	6 (8.2)	13 (26.0)	
Clinical M category, n (%)				0.962
M0	106 (86.2)	63 (86.3)	43 (86.0)	
M1	17 (13.8)	10 (13.7)	7 (14.0)	

Data are presented as mean ± SD or n (%). *p* values were calculated using the independent-samples *t* test (Welch’s correction when variances were unequal) for continuous variables and Pearson’s χ^2^ test for categorical variables; Fisher’s exact test was used where appropriate. For multi-level categorical variables with sparse cells (expected count <5), χ^2^-based *p-* values should be interpreted with caution; therefore, sleep status was additionally analyzed as a binary variable (normal vs any sleep problem) for robustness.

### 3.2 Longitudinal nutritional changes and trajectory modeling

**Figure 1:**
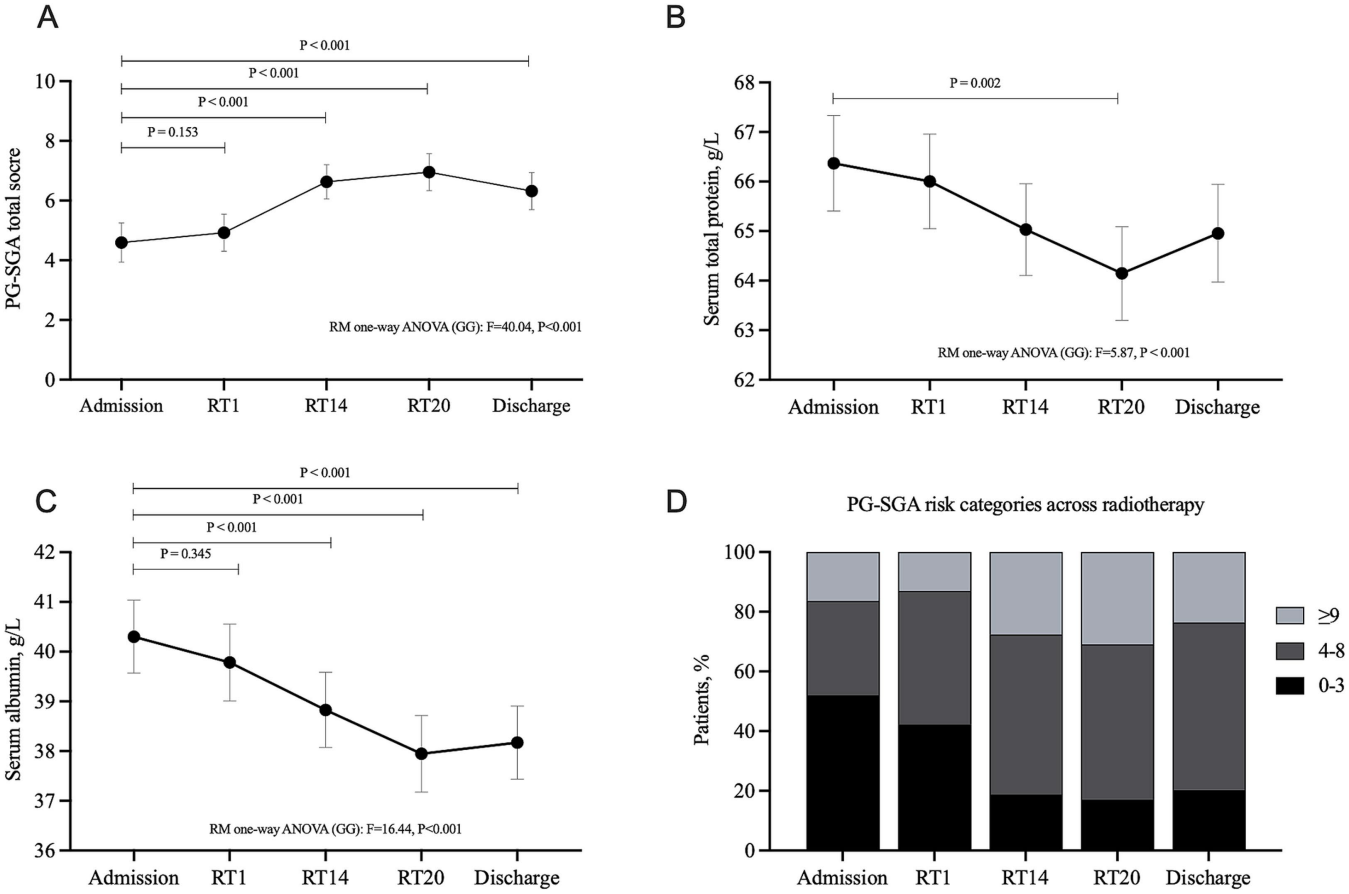
Longitudinal trajectories and distribution of nutritional status during radiotherapy. **(A)** PG-SGA trajectory across radiotherapy. **(B)** Longitudinal changes in serum total protein. **(C)** Longitudinal changes in serum albumin. **(D)** Distribution of PG-SGA risk categories across radiotherapy. Error bars in **(A–C)** represent 95% confidence intervals (CIs). Longitudinal changes in **(A–C)** were analyzed using repeated-measures ANOVA with Greenhouse–Geisser correction.

PG-SGA scores increased significantly over the RT course, rising from admission (4.59 ± 3.66) to RT14 (6.63 ± 3.20) and peaking at RT20 (6.95 ± 3.48), remaining above baseline at discharge (*p* < 0.001; Figure 1A). The distribution of risk categories shifted toward higher-risk strata over time (Figure 1D). Concordantly, serum albumin declined from admission (40.30 ± 4.11 g/L) to RT20 (37.95 ± 4.31 g/L; *p* < 0.001; Figure 1C), while total protein showed a modest decrease (*p* < 0.001; Figure 1B). All five assessments were completed for all patients (Table 2); body weight and BMI trajectories are reported in Supplementary Table S2.

**Table 2:** Longitudinal changes in PG-SGA score and serum nutritional biomarkers during radiotherapy.

Variable	Admission	RT1	RT14	RT20	Discharge
PG-SGA total score	4.59 ± 3.66	4.92 ± 3.47	6.63 ± 3.20	6.95 ± 3.48	6.32 ± 3.50
Total protein, g/L	66.37 ± 5.40	66.00 ± 5.34	65.03 ± 5.19	64.15 ± 5.30	64.96 ± 5.52
Albumin, g/L	40.30 ± 4.11	39.78 ± 4.33	38.83 ± 4.23	37.95 ± 4.31	38.17 ± 4.13

Values are mean ± SD (*n* = 123 at each timepoint).

To further characterize repeated-measures trajectories, linear mixed-effects modeling was performed using PG-SGA across the five prespecified timepoints. A significant time effect was observed (*F* = 25.176, *p* < 0.001), supporting longitudinal change in nutritional status during radiotherapy. Baseline NRS 2002 score was also significantly associated with longitudinal PG-SGA burden (*F* = 46.601, p < 0.001), whereas baseline sleep problems (*F* = 0.262, *p* = 0.610), N3 nodal stage (*F* = 0.591, *p* = 0.443), and age (*F* = 0.438, *p* = 0.509) were not significant overall main effects; chronic disease showed a borderline association (*F* = 3.797, *p* = 0.054).

In an interaction model, the time effect remained significant. No significant time-by-sleep interaction was observed (*F* = 1.403, *p* = 0.232). A significant time-by-N3 interaction was identified (*F* = 2.590, *p* = 0.036), indicating that patients with advanced nodal disease followed a distinct nutritional trajectory over the radiotherapy course (Supplementary Figure S1).

### 3.3 Predictors of nutritional deterioration and sensitivity analyses

In univariable logistic regression (Supplementary Table S3), sleep problems (OR 2.213, 95% CI 1.020–4.798; *p* = 0.044) and N3 nodal disease (OR 3.924, 95% CI 1.378–11.175; *p* = 0.011) were associated with higher odds of ND.

**Figure 2:**
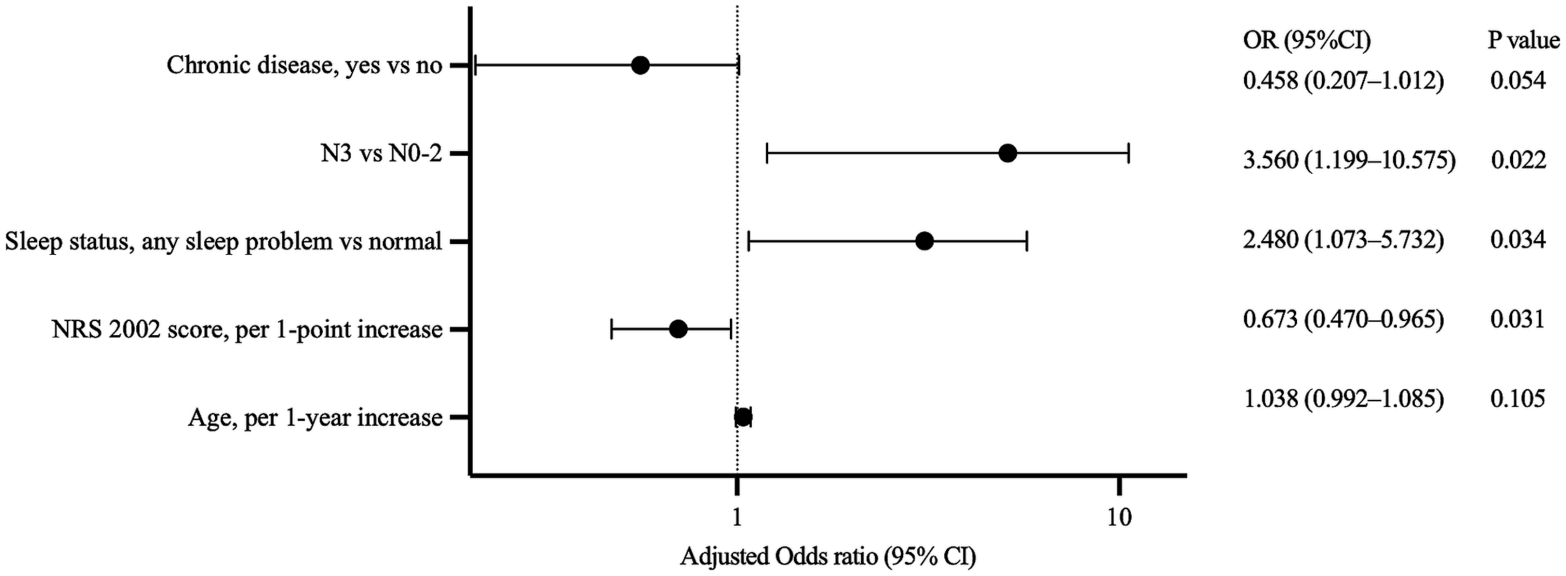
Forest plot of multivariable logistic regression model for the primary categorical endpoint of nutritional deterioration, showing adjusted odds ratios (aORs) and 95% confidence intervals (CIs). The dotted vertical line indicates OR = 1. ORs are adjusted for age, NRS 2002 score, sleep status, nodal stage, and chronic disease. Variable labels indicate the comparison or unit of increase where applicable (e.g., per 1-point increase for continuous score variables). Estimates were derived from multivariable binary logistic regression. *n* = 123.

In the multivariable model (Figure 2), sleep problems remained independently associated with ND (aOR 2.480, 95% CI 1.073–5.732; *p* = 0.034), alongside N3 disease (aOR 3.560, 95% CI 1.199–10.575; *p* = 0.022). Conversely, a higher baseline NRS 2002 score was associated with lower odds of ND under this specific triage-worsening definition (aOR 0.673; *p* = 0.031).

To complement the categorical endpoint, continuous change in PG-SGA from admission to discharge was additionally analyzed (∆PG = discharge PG-SGA − admission PG-SGA). In unadjusted comparisons, patients with baseline sleep problems showed numerically greater PG-SGA worsening than those without sleep problems (2.13 ± 3.03 vs. 1.54 ± 3.24), although this difference was not statistically significant (*p* = 0.337). Patients with N3 disease had significantly greater worsening than those with N0–2 disease (3.26 ± 3.57 vs. 1.44 ± 3.03; *p* = 0.021). In multivariable linear regression, N3 disease remained independently associated with greater PG-SGA worsening (*B* = 1.582, 95% CI 0.056–3.108; *p* = 0.042), while a higher baseline NRS 2002 score was associated with a smaller ∆PG change (*B* = −0.583, 95% CI − 1.049 to −0.118; *p* = 0.014). The association for baseline sleep problems remained directionally positive but was attenuated and no longer statistically significant (*B* = 0.619, 95% CI − 0.564 to 1.803; *p* = 0.302). Detailed results of the continuous-change analyses are provided in Supplementary Table S4.

Sensitivity analysis excluding patients with severe baseline nutritional status (PG-SGA ≥ 9) supported the robustness of the N3 association and suggested potential baseline constraint in the categorical endpoint. Advanced nodal stage remained independently associated with ND (OR 3.284, 95% CI 1.031–10.461; *p* = 0.044), while the association for sleep problems persisted directionally but was attenuated to borderline significance (OR 2.383, 95% CI 0.971–5.846; *p* = 0.058) (Supplementary Figure S2). The inverse association observed for baseline NRS 2002 score in the primary model was no longer evident in the restricted sample (OR 0.917, 95% CI 0.584–1.440; *p* = 0.706), further suggesting possible baseline constraint under the categorical endpoint definition.

### 3.4 Exploratory sleep-nutrition associations

**Figure 3:**
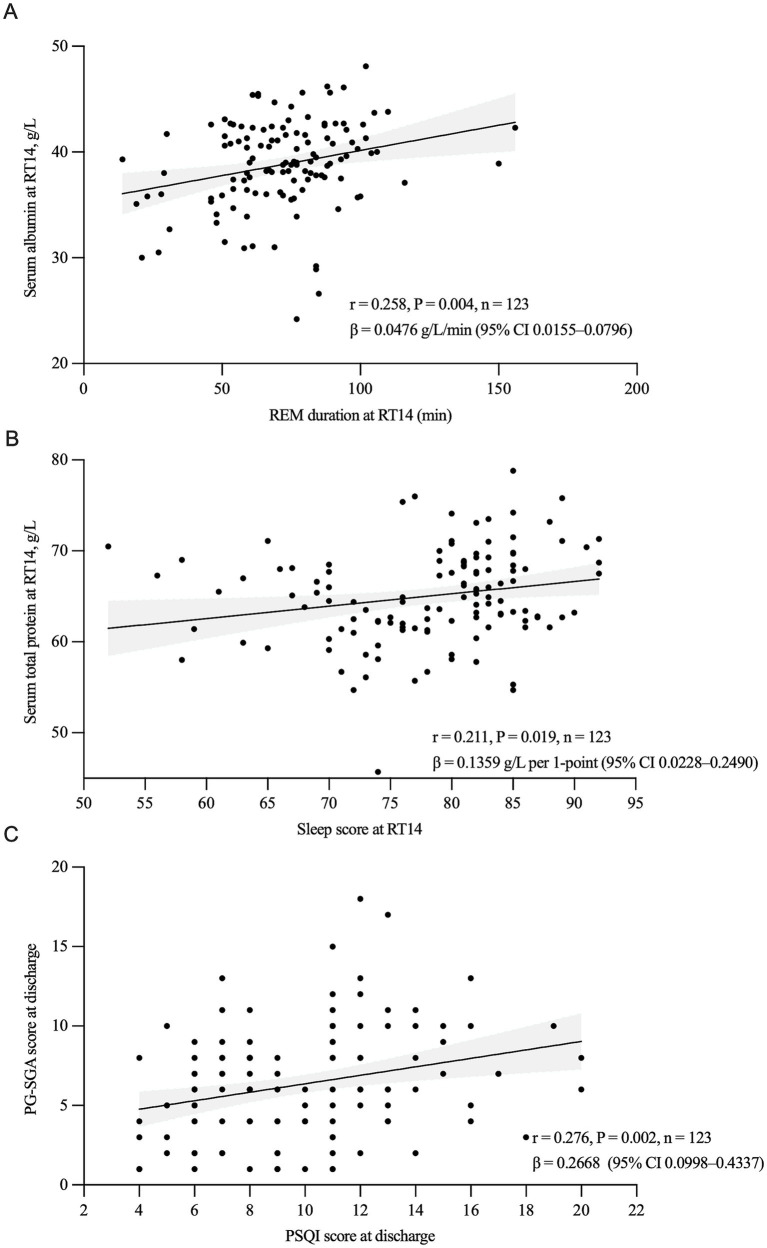
Exploratory associations between sleep metrics and nutritional indicators. **(A)** Wearable-derived REM duration at RT14 was positively associated with serum albumin at RT14. **(B)** Wearable-derived sleep score at RT14 was positively associated with serum total protein at RT14. **(C)** PSQI score at discharge was positively associated with PG-SGA score at discharge. Associations were assessed using Pearson correlation analysis.

Exploratory analyses linked sleep features to nutritional indicators. At RT14, wearable-derived REM duration correlated positively with serum albumin (*r* = 0.258, *p* = 0.004; Figure 3A), and sleep score correlated with total protein (*r* = 0.211, *p* = 0.019; Figure 3B). At discharge, higher PSQI scores (worse sleep quality) were associated with higher PG-SGA scores (worse nutrition) (*r* = 0.276, *p* = 0.002; Figure 3C).

### 3.5 Prototype development: esophageal care companion

**Figure 4:**
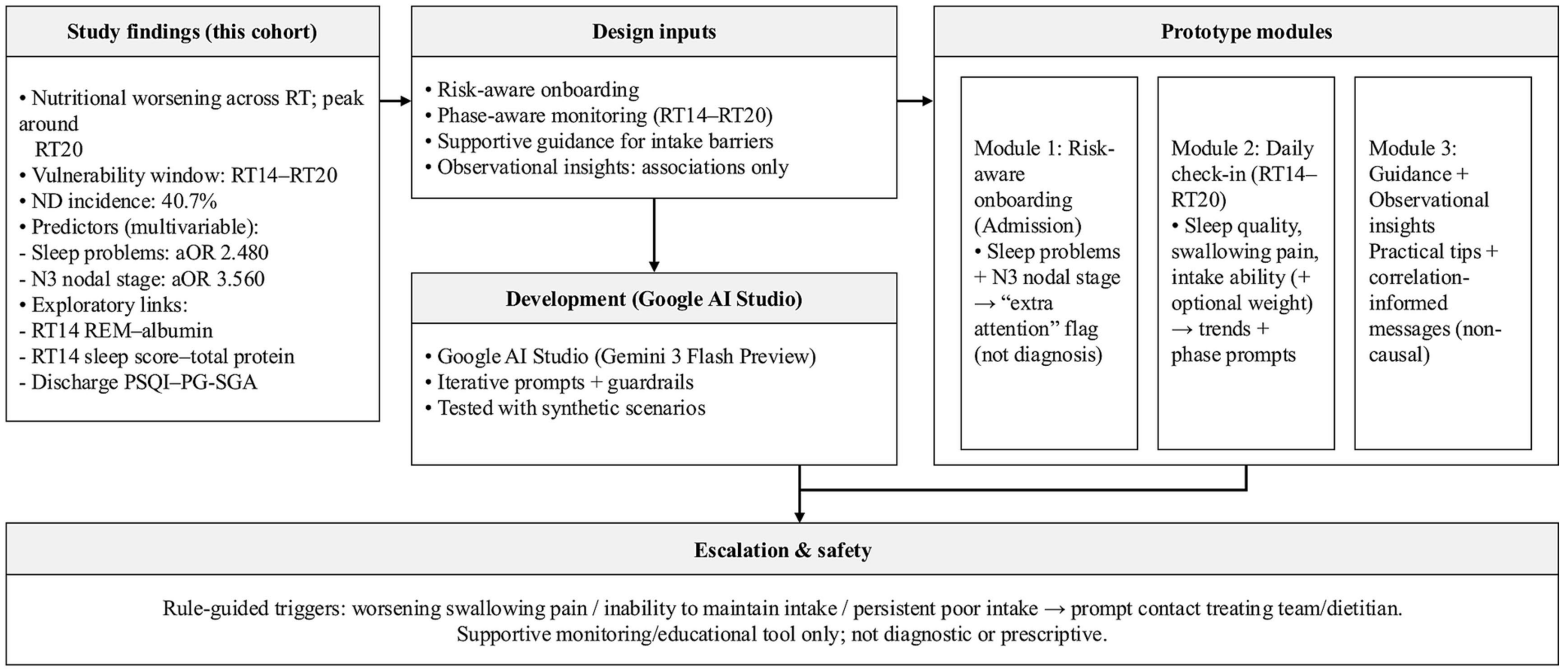
Evidence-to-prototype translation framework for the esophageal care companion. The diagram summarizes how cohort findings informed risk-aware onboarding, phase-specific monitoring (RT14–RT20), and supportive guidance with non-causal observational insights, with rule-guided escalation prompts for concerning symptoms.

We developed a patient-facing prototype structured around admission risk flags and the observed vulnerability window (Figure 4). The onboarding module screens for baseline sleep problems and nodal stage. Recognizing that nutritional status worsened markedly after fraction 14, the home dashboard prompts intensified daily tracking of sleep quality, swallowing pain, and intake ability during the RT14–RT20 window. The prototype provides correlation-informed “Observational Insights” based on cohort findings and translates phase-aware monitoring into a digital supportive-care workflow.

## 4 Discussion

In this prospective longitudinal cohort, we characterized time-resolved nutritional trajectories during esophageal cancer radiotherapy. Three major findings emerged. First, nutritional status worsened over the course of radiotherapy, with PG-SGA peaking around the 20th fraction and concordant declines in serum biomarkers. Second, approximately two in five patients experienced categorical nutritional deterioration (ND) by discharge. Third, complementary analyses suggested that advanced nodal stage (N3) was the most robust marker of a less favorable nutritional course, while baseline sleep problems retained potential clinical relevance as a low-burden observational signal.

A key contribution of this study is the delineation of a clinically interpretable vulnerability window. The synchronized worsening of PG-SGA with declines in albumin supports that nutritional compromise accelerates as cumulative treatment burden develops, highlighting RT14–RT20 as a practical period for intensified surveillance. This interpretation is also supported by longitudinal modeling, which confirmed a significant time effect across repeated PG-SGA assessments. The timing is consistent with the natural history of acute radiation esophagitis during radiotherapy, as symptoms most commonly begin within the second to third week after treatment initiation (27, 28). In routine practice, these findings support phase-aware nutritional reassessment around the second or third treatment week, in line with guidance emphasizing ongoing monitoring during anticancer therapy (8).

Within this framework, advanced nodal stage emerged as the most statistically robust high-risk feature across complementary analyses. N3 disease was associated with the primary categorical ND endpoint, greater admission-to-discharge PG-SGA worsening, and a significant time-by-nodal-stage interaction in longitudinal modeling. Together, these findings suggest that the impact of nodal burden is not limited to a higher static risk, but may also be reflected in a distinct and less favorable nutritional trajectory over the course of radiotherapy. A plausible explanation is that more advanced nodal disease may require larger treatment fields or be associated with greater treatment-related toxicity, thereby amplifying swallowing-related symptom burden and nutritional compromise.

Baseline sleep problems may still have clinical relevance in this study, particularly because they are easy to assess, low cost, noninvasive, and readily applicable in supportive care. In the primary categorical model, baseline sleep problems were associated with higher odds of ND, and exploratory analyses suggested phase-specific links between sleep-related measures and nutritional indicators. Although this signal was less consistent in complementary continuous-change and longitudinal models, sleep remains of interest because it may capture aspects of symptom burden or functional vulnerability not fully reflected by tumor stage alone (29, 30). In this sense, sleep disturbance may be viewed as a pragmatic observational signal that could help support baseline risk assessment in routine care (29, 31).

Exploratory analyses provided convergent signals linking sleep measures to nutritional indicators across multiple treatment phases (32). These findings may reflect broader symptom burden, functional impairment, or systemic stress during treatment rather than a direct causal pathway (29, 32). Emerging evidence also suggests that inflammatory and immune-microenvironmental processes may contribute to disease-related vulnerability in upper aerodigestive tract malignancies, although these mechanisms were not directly evaluated in our cohort (33). Future studies should further clarify whether sleep disturbance precedes worsening nutrition, tracks with concurrent symptom burden, or simply co-occurs with it during treatment.

Translating these findings, our “Esophageal Care Companion” prototype demonstrates how to operationalize phase-aware monitoring within a digital supportive-care framework. By aligning self-monitoring with the RT14–RT20 vulnerability window, such tools could help reduce delays in escalating supportive measures (e.g., texture modification, analgesia, dietitian referral) as toxicities accumulate (34). In our cohort, nutritional support during treatment most commonly involved oral nutritional supplementation and diet modification or texture adjustment, while more intensive enteral or parenteral support was used in a smaller subset of patients. In this context, baseline disease burden and low-burden observational signals such as sleep may be incorporated as part of supportive-care monitoring (29).

Although caloric restriction or fasting-mimicking strategies are being investigated in selected oncologic contexts, these approaches differ conceptually from the unintended reduction in oral intake and treatment-related nutritional deterioration evaluated in the present study. In our cohort, worsening nutritional status occurred in the setting of radiotherapy-related symptom burden and declining nutritional indicators, and was therefore more relevant to supportive-care risk than to intentional metabolic intervention (35).

This study has several limitations. It was a single-center study with a modest sample size. The categorical ND endpoint was selected for clinical interpretability, but it may be influenced by baseline severity constraints and may not fully capture deterioration magnitude; this concern was partly addressed through complementary continuous-change analysis, exclusion-based sensitivity analysis, and longitudinal mixed-effects modeling. Sleep-related associations were not fully consistent across complementary analyses and should therefore be interpreted cautiously. Residual confounding by symptom burden or other unmeasured clinical factors remains possible. In addition, wearable sleep metrics were derived from a consumer device rather than polysomnography, necessitating cautious interpretation.

## 5 Conclusion

Nutritional risk during esophageal cancer radiotherapy worsens in a time-dependent manner, with a clinically relevant mid-to-late treatment vulnerability window around RT14–RT20. Under the prespecified categorical PG-SGA endpoint, nearly 40% of patients experienced deterioration by discharge. Advanced nodal stage was the most robust marker of a less favorable nutritional trajectory, whereas baseline sleep problems retained potential clinical relevance as an easily assessable, low-burden observational signal. These findings support phase-aware monitoring during radiotherapy and suggest that simple baseline signals, including sleep status, may help inform proactive supportive care, including earlier texture modification, analgesia to facilitate oral intake, oral nutritional supplementation, dietitian referral, and timely escalation to enteral or parenteral nutrition when clinically indicated.

## Data Availability

The original contributions presented in the study are included in the article/Supplementary material, further inquiries can be directed to the corresponding authors.
