# Supplementary material for: Dynamic nutritional trajectories and deterioration risk in esophageal cancer radiotherapy: a prospective study
Source: Front Nutr. 2026 Jun 18;13:1828784. doi: 10.3389/fnut.2026.1828784 (PMC13329822; doi:10.3389/fnut.2026.1828784)

**Supplementary Table S1. Radiotherapy regimen and supportive care during treatment (n = 123).**

| **Variable** | **n (%)** |
| --- | --- |
| **Part A. Radiotherapy regimen** |  |
| 50 Gy / 25 fractions / 2 Gy per fraction | 67 (54.5) |
| 60 Gy / 30 fractions / 2 Gy per fraction | 23 (18.7) |
| 40 Gy / 20 fractions / 2 Gy per fraction | 13 (10.6) |
| 45 Gy / 25 fractions / 1.8 Gy per fraction | 6 (4.9) |
| 50.4 Gy / 28 fractions / 1.8 Gy per fraction | 4 (3.3) |
| Other regimens | 10 (8.1) |
| **Part B. Nutritional support** |  |
| Any nutritional support | 99 (80.5) |
| Oral nutritional supplementation (ONS) | 90 (73.2) |
| Nutrition consultation | 9 (7.3) |
| Diet modification or texture adjustment | 94 (76.4) |
| Nasogastric tube feeding | 23 (18.7) |
| Post-pyloric tube feeding | 5 (4.1) |
| Gastrostomy | 1 (0.8) |
| Parenteral nutrition | 34 (27.6) |
| **Part C. Related supportive care** |  |
| Analgesic support to facilitate oral intake | 53 (43.1) |

Data are presented as n (%). Percentages were calculated using the full cohort (N = 123). Analgesic support to facilitate oral intake was recorded as a related supportive-care measure and was not counted as nutritional support.

**Supplementary Table S2. Changes in body weight and BMI during radiotherapy.** Values are mean ± SD.

| Timepoint | Weight, kg | BMI, kg/m² | n |
| --- | --- | --- | --- |
| Admission | 57.46 ± 11.20 | 21.35 ± 3.27 | 123 |
| RT14 | 56.90 ± 11.01 | 21.16 ± 3.28 | 123 |
| RT20 | 56.62 ± 11.00 | 21.05 ± 3.27 | 123 |
| Discharge | 56.43 ± 10.97 | 20.98 ± 3.29 | 123 |

**Supplementary Table S3. Univariable logistic regression for nutritional deterioration (ND) (n = 123).**

ND was coded as 1 (deterioration) and 0 (stable/improved). ORs were derived from univariable binary logistic regression. Reference categories: Female, No chronic disease, Normal sleep, N0-2, M0, No concurrent chemotherapy. For continuous variables, ORs represent the change in odds per 1-unit increase.

| Variable | Comparison | OR (95% CI) | P value |
| --- | --- | --- | --- |
| Sex | Male vs Female | 1.214 (0.504-2.927) | 0.665 |
| Chronic disease | Yes vs No | 0.471 (0.226-0.983) | 0.045 |
| Sleep status (binary) | Any sleep problem vs normal | 2.213 (1.020-4.798) | 0.044 |
| N stage (binary) | N3 vs N0-2 | 3.924 (1.378-11.175) | 0.011 |
| M stage | M1 vs M0 | 1.026 (0.362-2.903) | 0.962 |
| Concurrent chemotherapy | Yes vs No | 0.917 (0.445-1.889) | 0.813 |
| Age | Per 1-year increase | 1.022 (0.983-1.063) | 0.261 |
| NRS 2002 score | Per 1-point increase | 0.742 (0.539-1.021) | 0.067 |

**Supplementary Table S4. Continuous-change analysis of PG-SGA worsening (ΔPG).**

**Part A. Unadjusted comparisons of ΔPG by baseline sleep status and nodal stage**

| Variable | Group | n | ΔPG mean ± SD | P value |
| --- | --- | --- | --- | --- |
| Baseline sleep status | Normal | 84 | 1.54 ± 3.24 | 0.337 |
|  | Any sleep problem | 39 | 2.13 ± 3.03 |  |
| Nodal stage | N0-2 | 104 | 1.44 ± 3.03 | 0.021 |
|  | N3 | 19 | 3.26 ± 3.57 |  |

**Part B. Multivariable linear regression for ΔPG**

| Predictor | B | 95% CI | P value |
| --- | --- | --- | --- |
| Age (per 1-year increase) | 0.023 | -0.036 to 0.082 | 0.447 |
| NRS 2002 score (per 1-point increase) | -0.583 | -1.049 to -0.118 | 0.014 |
| Chronic disease (yes vs no) | -0.550 | -1.657 to 0.558 | 0.328 |
| Sleep status (any sleep problem vs normal) | 0.619 | -0.564 to 1.803 | 0.302 |
| N3 nodal stage (vs N0-2) | 1.582 | 0.056 to 3.108 | 0.042 |

**Abbreviations**: ΔPG, discharge PG-SGA minus admission PG-SGA; PG-SGA, Patient-Generated Subjective Global Assessment; CI, confidence interval; SD, standard deviation; NRS 2002, Nutritional Risk Screening 2002.

**Notes**: Part A shows unadjusted group comparisons using independent-samples t tests. Part B shows multivariable linear regression including age, NRS 2002 score, chronic disease, baseline sleep problems, and advanced nodal stage (N3 vs N0–2). Positive B values indicate greater PG-SGA worsening from admission to discharge.

**Model summary**: R² = 0.104, adjusted R² = 0.066; overall model F = 2.727, P = 0.023.

**Supplementary Figure S1. Estimated marginal mean PG-SGA trajectories stratified by nodal stage.** Estimated marginal means of PG-SGA total score across the five prespecified radiotherapy timepoints are shown for patients with N0–2 and N3 disease from the linear mixed-effects interaction model. Error bars represent 95% confidence intervals. The significant time-by-N3 interaction (P = 0.036) is reflected by the differing longitudinal trajectories.


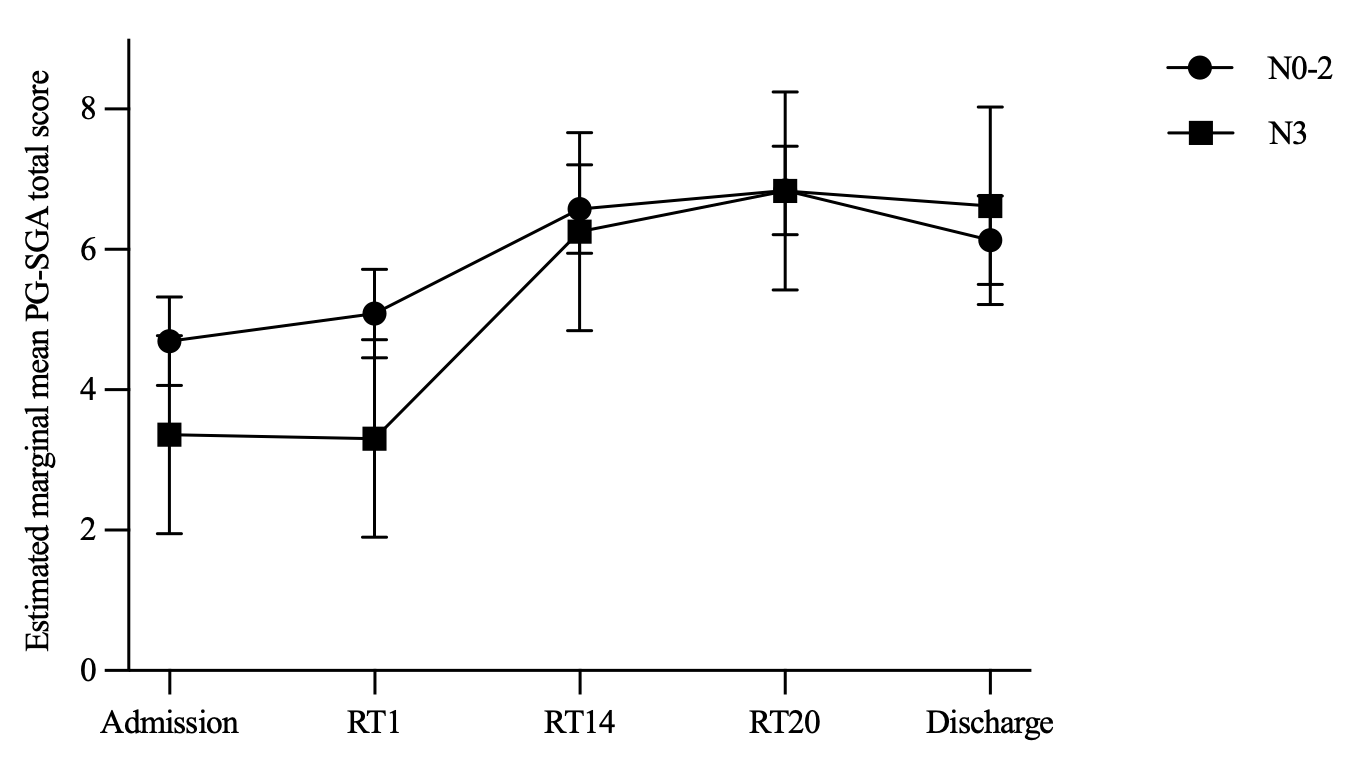


**Supplementary Figure S2. Forest plot of multivariable logistic regression for nutritional deterioration (ND) after exclusion of patients with baseline PG-SGA ≥ 9.** Adjusted odds ratios (aORs) and 95% confidence intervals (CIs) are shown. The dotted vertical line indicates OR = 1. ORs are adjusted for age, NRS 2002 score, sleep status, nodal stage, and chronic disease. n = 103.


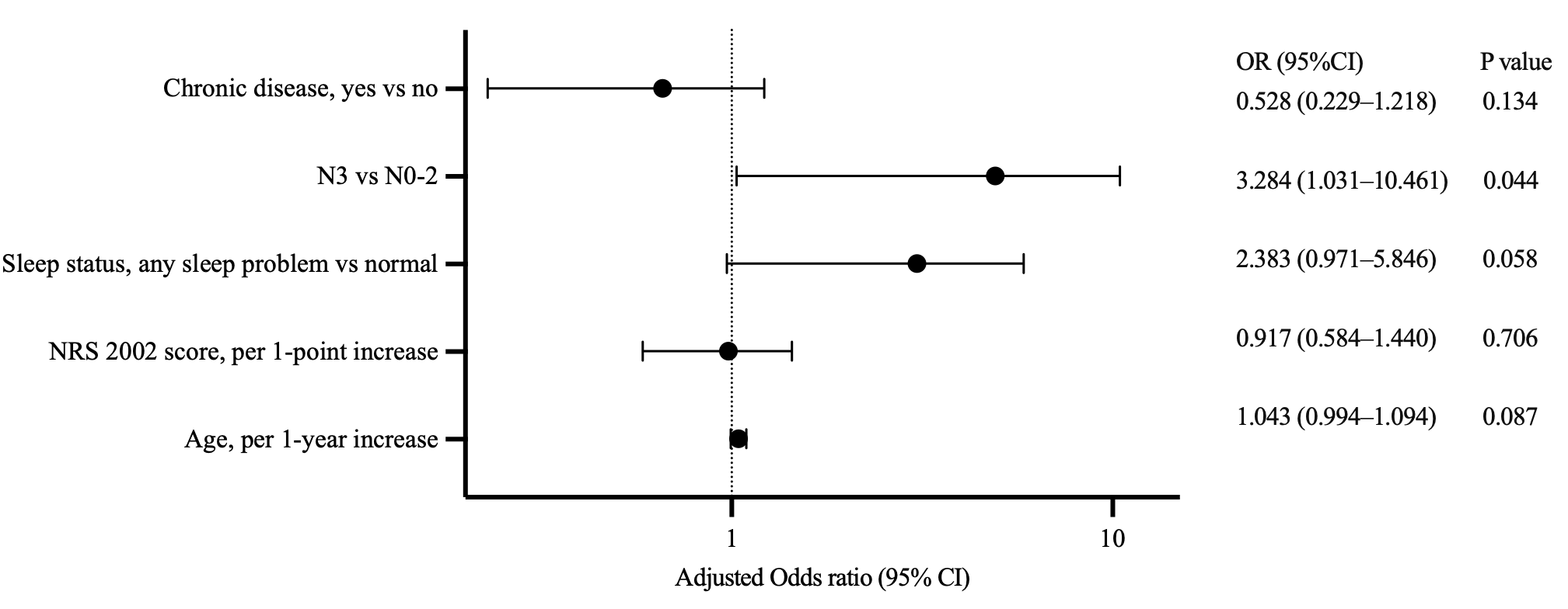

Supplement: Supplementary file 1 [file Table_1.docx]
